# Diet enriched in saturated fatty acids alters mean arterial blood pressure and fat metabolism but not autonomic balance in rats

**DOI:** 10.1590/1414-431X2026e15215

**Published:** 2026-09-07

**Authors:** T.H.G. Rodrigues, A.P.F.C. Arcoverde-Mello, A.C. Simões-Alves, G.M. da Silva, T.R.L.A. Lima, M.P. Fernandes, V.O. Nogueira, A.G. Wanderley, J.H. Costa-Silva

**Affiliations:** 1 Laboratório de Nutrição, Atividade Física e Plasticidade Fenotípica, Universidade Federal de Pernambuco, Vitória de Santo Antão, PE, Brasil; 2 Laboratório de Bioquímica Geral, Molecular e do Exercício, Universidade Federal de Pernambuco, Vitória de Santo Antão, PE, Brasil; 3 Departamento de Ciências Farmacêuticas, Universidade Federal de São Paulo, Diadema, SP, Brasil

**Keywords:** Autonomic control, Hypertension, Metabolic changes, Oxidative stress, Saturated fatty acid

## Abstract

This study aimed to evaluate the effects of a diet rich in saturated fatty acids on fat metabolism and oxidative damage and its repercussions on arterial blood pressure and autonomic balance. During pregnancy and lactation, primiparous Wistar rats received a standard laboratory diet. After weaning, the pups were divided into two groups according to their diet: a normolipidic diet (Control group, C) and a high-fat diet (Hyperlipidic group, HL). Food consumption, weight development, serum biochemical parameters, arterial blood pressure, and heart rate were evaluated. Adipose tissue was collected for oxidative stress measurements at 90 days of life (d). The HL group had a greater intake of lipids, greater abdominal circumference at 30 d, and increased oxidative stress in adipose tissue. Epididymal adipose tissue showed increased activity of fatty acid synthase (FAS) and decreased activity of β-hydroxyacyl-CoA dehydrogenase (β-HAD). These effects were associated with high mean arterial pressure but were not associated with changes in cardiovascular variability in time and frequency domains at 90 days. Thus, the intake of a diet rich in saturated fatty acids for up to 90 days may be related to a greater induction of oxidative stress in visceral adipose tissue, preceding the development of autonomic dysfunction and arterial hypertension in older individuals.

## Introduction

Since the mid-20th century, demographic, socioeconomic, and epidemiological changes have driven a global nutritional transition characterized by increased consumption of processed foods rich in saturated fats and reduced physical activity (1,2). In Brazil, the consumption of high-fat foods, characterized by a dietary pattern known as the “Western diet”, is common among families. This type of diet, prevalent in both developed and developing countries, is associated with increased body weight and accumulation of visceral adipose tissue, which are closely linked to cardiometabolic complications (3,4).

Studies in experimental models indicate that diets rich in saturated fats promote alterations in lipid metabolism, leading to mitochondrial dysfunction and overproduction of reactive oxygen species (ROS), resulting in oxidative stress, reduced antioxidant defenses, and arterial hypertension, among other cardiometabolic disorders (5). This imbalance manifests itself through the activation of multiple cellular pathways, including persistent inflammatory activation, characterized by elevated levels of specific cytokines, such as tumor necrosis factor (TNF)-α, interleukin (IL)-6, and IL-1β (5).

This pro-oxidant and inflammatory environment contributes to the release of adipokines and cytokines that alter endothelial signaling and increase sympathetic activity. As a consequence, there is an increase in blood pressure and autonomic imbalance, characterized by sympathetic hyperactivity and a reduction in parasympathetic tone, affecting mainly cardiac modulation (6).

In humans, diets rich in saturated fats contribute to dyslipidemia, altered lipid profiles, and accumulation of visceral adipose tissue, especially epididymal, aggravating the risk of metabolic diseases (7). Despite this evidence, few studies have explored in detail the lipid composition of these diets and their relationship with the actual consumption patterns of the population.

On the basis of this context, we hypothesized that prolonged exposure to a high-fat diet induces metabolic and cardiovascular dysfunctions, promotes oxidative stress, impairs autonomic regulation, and alters lipid homeostasis. Therefore, the objective of this study was to evaluate the effects of a diet enriched with saturated fatty acids on lipid metabolism, oxidative stress in adipose tissue, blood pressure, and autonomic balance in adult Wistar rats.

## Material and Methods

### Ethics

This work was approved by the Ethics Committee for Animal Use (CEUA-UFPE), under protocol No. 23076.046459/2018-17, and followed the norms and standards for good practices in the handling of laboratory animals.

### Animals

Primiparous albino female Wistar rats (n=20), weighing over 200 g and 90 days old, from the Bioterium of the Vitória Academic Center (CAV) of the Federal University of Pernambuco, were used. The rats were mated with fertile males in a 3:1 ratio and, after confirmation of pregnancy by observing the presence of spermatozoa in the vaginal smear, were transferred to individual cages and received standard laboratory diet. The offspring of each female were standardized to 8 pups per litter, prioritizing the maintenance of males. In cases where the litter had fewer than 8 males, females were used exclusively to standardize the litter size until weaning. The animals were kept at room temperature of 22±1°C, with a controlled light-dark cycle (light on from 6 pm to 6 am) and received a standard laboratory diet until the end of lactation. After weaning (21st postnatal day), male pups from different litters were grouped and randomly assigned to the Control group (C-normolipidic) or to the hyperlipidic group (HL), with balanced representation of multiple litters to minimize litter effects.

### Diets

The control diet was prepared according to the guidelines of the American Institute of Nutrition (8), and the hyperlipidic diet was prepared as previously described (9), with adaptations in ingredients. The macronutrient composition of the diets was as follows (in g/100 g): Control diet contained 19% lipids (17.32% saturated fatty acids, 29.93% monounsaturated, and 52.75% polyunsaturated), 20% protein, and 61% carbohydrates, with an energy density of 3.69 kcal/100 g. The hyperlipidic diet contained 31% lipids (29.59% saturated fatty acids, 34.77% monounsaturated, and 35.64% polyunsaturated), 20% protein, and 49% carbohydrates, with an energy density of 4.46 kcal/100 g. Diets were weighed per cage and offered daily, as well as tailings, to quantify daily food consumption.

### Body weight

The animals' body weight was monitored daily from birth to weaning (21 days of age) and then every 10 days until 90 days of age, using an appropriate scale (model AS-1000, Marte; Brazil), with a margin of error of 0.01 g (10). Weight gain was calculated in the periods from 21 to 30 d and from 30 to 90 d. Abdominal circumference, tail length, and naso-anal length were measured weekly from birth to day 90. After obtaining these measurements, the Lee index was calculated on day 90 from the ratio of the cubic root of body weight to the animal's naso-anal length (11). The energy efficiency ratio (EER) was calculated as the ratio between body mass gain and total energy intake over the same experimental period, according to the following formula: EER (%) = (mean body mass gain × 100) / total energy intake, as previously described (12), for the periods from 21 to 30 days and from 30 to 90 days.

### Serum analyses

Biochemical parameters were analyzed in serum collected through the caudal vein of animals anesthetized with ketamine (80 mg/kg, *ip*) and xylazine (10 mg/kg, *ip*) after fasting for 6 h at 21, 30, and 90 d. Blood collections were performed in the morning and between 1:00 and 3:00 PM. After collection, blood samples were placed in tubes without anticoagulants and centrifuged at 1300 *g* for 10 min at room temperature (23°C) to obtain serum. The supernatant was collected with a pipette and transferred to an Eppendorf tube for analysis. Serum albumin, total protein, total cholesterol, triglycerides (TG), and glucose levels were analyzed using their respective standard laboratory kits (Labtest Diagnostico AS, Brazil). From the dosage of triglycerides, the value of very-low density lipoprotein (VLDL)-cholesterol was obtained by the formula of Friedewald=TG/5 (13).

### Arterial blood pressure

The analysis of cardiovascular parameters was performed through cannulation of the femoral artery to record the pulsatile pressure. Briefly, the surgical procedure was performed at 89 d. The animals were anesthetized with ketamine (80 mg/kg, *ip*) and xylazine (10 mg/kg, *ip*), and then the femoral artery was cannulated with the insertion of a polyethylene catheter as described previously (10). The cannula was positioned subcutaneously on the animal's back and exteriorized on the animal's back between the shoulder blades. The animals received a dose of anti-inflammatory ketoprofen (5 mg/kg, *im*) and were accommodated in individual boxes until the time of recording. Blood pressure was recorded 24 h after the surgical procedure, at 90 d, so that the animals could fully recover. Pulsed blood pressure was recorded for 60 min by connecting the femoral artery cannula to a mechanical pressure transducer and a signal amplifier (ML866/P/P, ADInstruments, Power Lab; Australia). The records were stored on a computer equipped with appropriate software (LabChart^©^ Pro, ADInstruments) and analyzed later. Mean arterial pressure and heart rate were derived from pulsatile blood pressure measurements, calculated by the acquisition and analysis system, as described (10).

### Spectral analysis of cardiovascular variability

Cardiovascular variability was obtained from blood pressure and heart rate through time and frequency domain spectral analysis with using Cardioseries (Cardioseries^©^ 2.4; Brazil) and HRV programs (LabChartPro^©^ 8.1, ADInstruments). The frequency domain assessment was performed on a 10-min stable pulsatile pressure record and generated a graph of spectral power density, which can be divided into the following components: high frequency (HF, waves between 0.75 and 3 Hz), low frequency (LF, waves between 0.2 and 0.75 Hz), and very low frequency (VLF, waves between 0.003 and 0.04 Hz); the LF/HF ratio was then calculated.

### Adipose tissue measurements

At 90 days, after cardiovascular recordings, the animals were anesthetized and euthanized by decapitation. After dissection, epididymal, retroperitoneal, mesenteric, and subcutaneous adipose tissues were carefully collected and weighed individually on a high-precision balance (0.001 g). Total body fat was determined by summing these adipose deposits. The adiposity index was calculated using the following formula: Adiposity index (%) = (total body fat / body mass) × 100 (14).

To evaluate the activity of the main lipid metabolism enzymes, antioxidant enzymes, and oxidative stress biomarkers, epididymal adipose tissue homogenate was prepared in an extraction buffer, followed by protein quantification (15). The enzymatic activity of β-hydroxyacyl-CoA dehydrogenase (β-HAD) was determined according to Ito et al. (16). Fatty acid synthase (FAS) activity was measured by monitoring the decrease in absorbance at 340 nm resulting from NADPH oxidation of malonyl-CoA after a 3-min incubation at 25°C in a FLUOstar Omega spectrofluorimeter (BMG Labtech, USA).

### Statistical analysis

Data are reported as means±SE. For comparison between the control group (C) and the hyperlipidic group (HL), Student's *t-*test and the Mann-Whitney test were used for parametric and non-parametric data, respectively. The level of significance was P<0.05. Data were analyzed using Graphpad Prism (GraphPad Software Corporation, version 7.0, 2007; USA).

## Results

**Figure 1:**
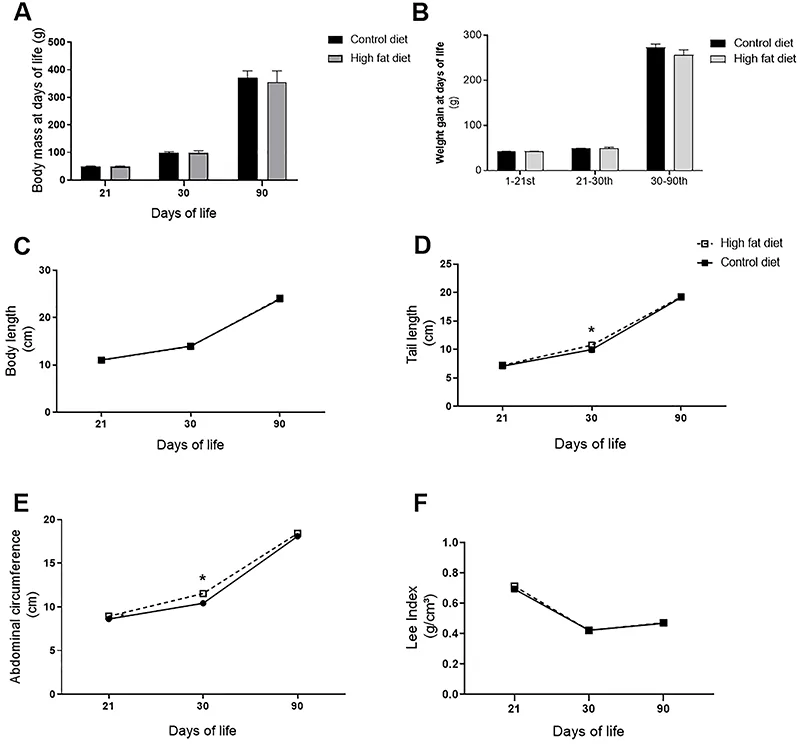
Anthropometric parameters measured at 21, 30, and 90 days of life of male Wistar rats under control and hyperlipidic diets at early ages. **A**, Body mass; **B**, weight gain; **C**, body length; **D**, tail length; **E**, abdominal circumference; **F**; Lee Index. Data are reported as means±SE (n=5-12). *P<0.05; unpaired Student's *t*-test.

The anthropometric results are shown in Figure 1. As shown in Figure 1A-C, the body mass, body weight gain, and body length of both groups were equivalent throughout growth with no significant differences. At 30 days of life, tail length, shown in Figure 1D, was shorter in the C group (C=9.99±0.21 *vs* HL=10.77±0.26 cm; P=0.03), and abdominal circumference, shown in Figure 1E, was greater in the HL group (C=10.43 ±0.17 *vs* HL=11.54±0.38 cm; P<0.0099). There was no difference in the other ages evaluated, as the Lee index (Figure 1F) did not differ between groups.

Food consumption at the ages of 22 to 30 days, 51 to 60 days, and 81 to 90 days is shown in Table 1. From 22 to 30 days, the total consumption of food, calories, and carbohydrates, but not proteins, was lower in the HL group than in the C group. The consumption of lipids was greater in the HL group at 22-30 days because the composition of the high-fat diet rich in saturated fatty acids remained high during the following periods. The EER significantly differed only during the initial period (22-30 days). The animals in the HL group continued to consume fewer carbohydrates and more lipids during the periods of 51-60 days and 81-90 days of life.

**Table 1:** Food consumption, calories, macronutrients, and the energy efficiency ratio in different periods of life of male Wistar rats submitted to control and hyperlipidic diets.

Parameters of food consumption	22-30 days		51-60 days		81-90 days	
	C	HL	C	HL	C	HL
Total consumption (g)	84.3±3.7	63.2±5.02*	183.7±6.6	169.4±7.9	191.3±11.3	183.4±8.3
Calories (kcal)	311±12.1	281.9 ±23.5*	678±24.3	755.6±35.4	705.7±41.9	818±37.1
Proteins (g)	15. 6±0.6	14.2±1.1	34.2±1.2	38.1±1.7	35.5±2.1	41.2±1.8
Carbohydrates (g)	57.8±2.2	34.5±2.8*	126.1±4.5	92.6±4.3*	131.2±7.7	100.3±4.5*
Lipids (g)	5.1±0.2	9. 6±0.8*	11.2±0.4	25.9±1.2*	11.6±0.6	28.1±1.2*
EER (g body weight gain/kcal)	0.58±0.02	0.71±0.01*	0.41±0.02	0.37±0.01	0.14±0.01	0.17±0.01

Data are reported as means±SE (n=5-12). *P<0.05 between groups in the same period; unpaired Student's *t*-test. EER: Energy efficiency ratio; C: Control group; HL: Hyperlipidic (diet rich in saturated fatty acids) group.

The high-fat diet did not induce changes in total protein, albumin, glucose, total cholesterol, VLDL, or TG levels at any age, as shown in Table 2.

**Table 2:** Serum biochemical parameters of male Wistar rats submitted to control diet (C) and hyperlipidic (HL) diet rich in saturated fatty acids.

Biochemical parameters	21 days		30 days		90 days	
	C	HL	C	HL	C	HL
Total proteins (g/dL)	5.4±0.2	5.9±0.2	4.7±0.1	4.9±0.1	6.2±0.1	6.04±0.1
Albumin (g/dL)	3.1±0.1	2.9±0.2	4.1±0.03	4.2±0.05	4.2±0.1	4.2±0.1
Glucose (mg/dL)	111.8±6.3	108±5.6	107.4±6.0	113.1±7.4	136.9±9.1	136.7±10.9
Cholesterol (mg/dL)	107±6.9	101.3±7.5	88.2±8.2	94.5±7.8	86.5±5.3	88.3±2.8
VLDL (mg/dL)	25.9±2.6	27.6±2.7	45.9±3.9	34.5±4.7	26.3±2.06	23.8±1.4
TG (mg/dL)	135.1±12.5	130.4±12.0	211.5±20.8	197.4±30.5	131.5±10.2	113.6±7.3

Data are reported as means±SE (n=5-12). P>0.05; unpaired Student's *t*-test. VLDL: very low-density lipoprotein; TG: triglycerides.

**Figure 2:**
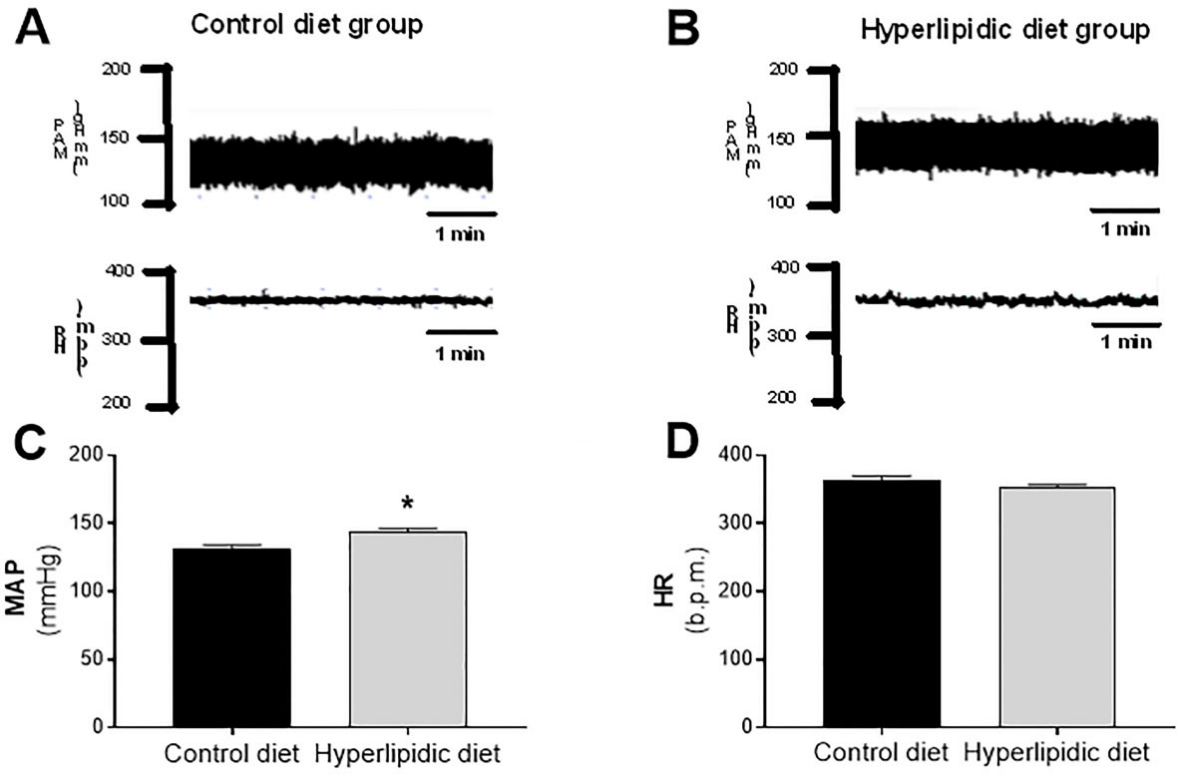
Cardiac function at 90 days in Wistar rats on control or hyperlipidic saturated fat diets. Representative tracings of mean arterial pressure (MAP) and heart rate (HR) of the control group and the hyperlipidic group (**A** and **B**) and the comparison between the groups (**C** and **D**). Data are reported as means±SE (n=4-5). *P<0.05; unpaired Student's *t*-test.

**Figure 3:**
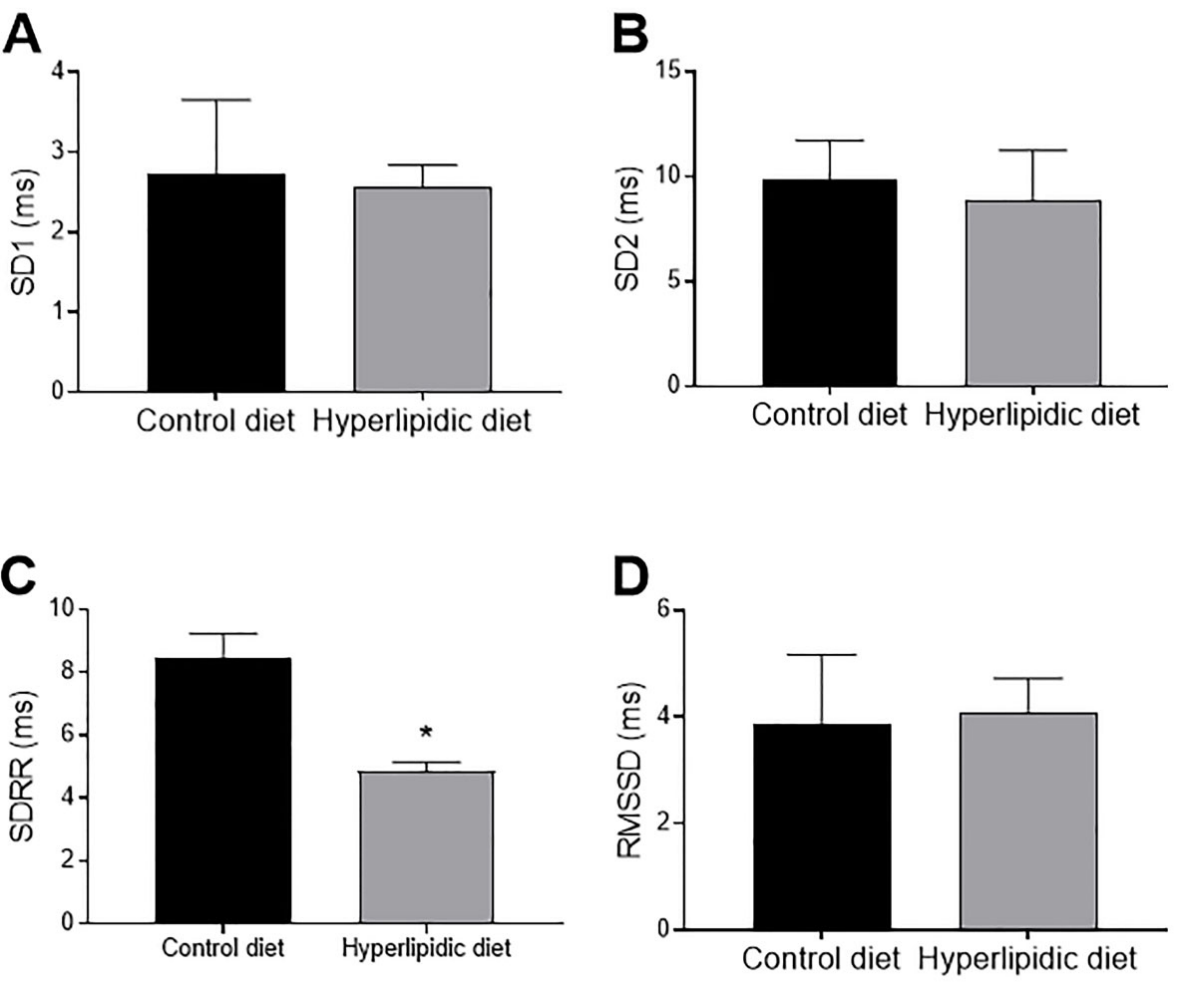
Time-domain cardiac variability in 90-day-old Wistar rats on control and high-fat diets. **A**, Short- and (**B**) long-term alterations; **C**, standard deviation of RR intervals (SDRR); **D**, root mean square successive difference (RMSSD). Data are reported as means±SE (n=4-5). *P<0.05; unpaired Student's *t*-test.

**Figure 4:**
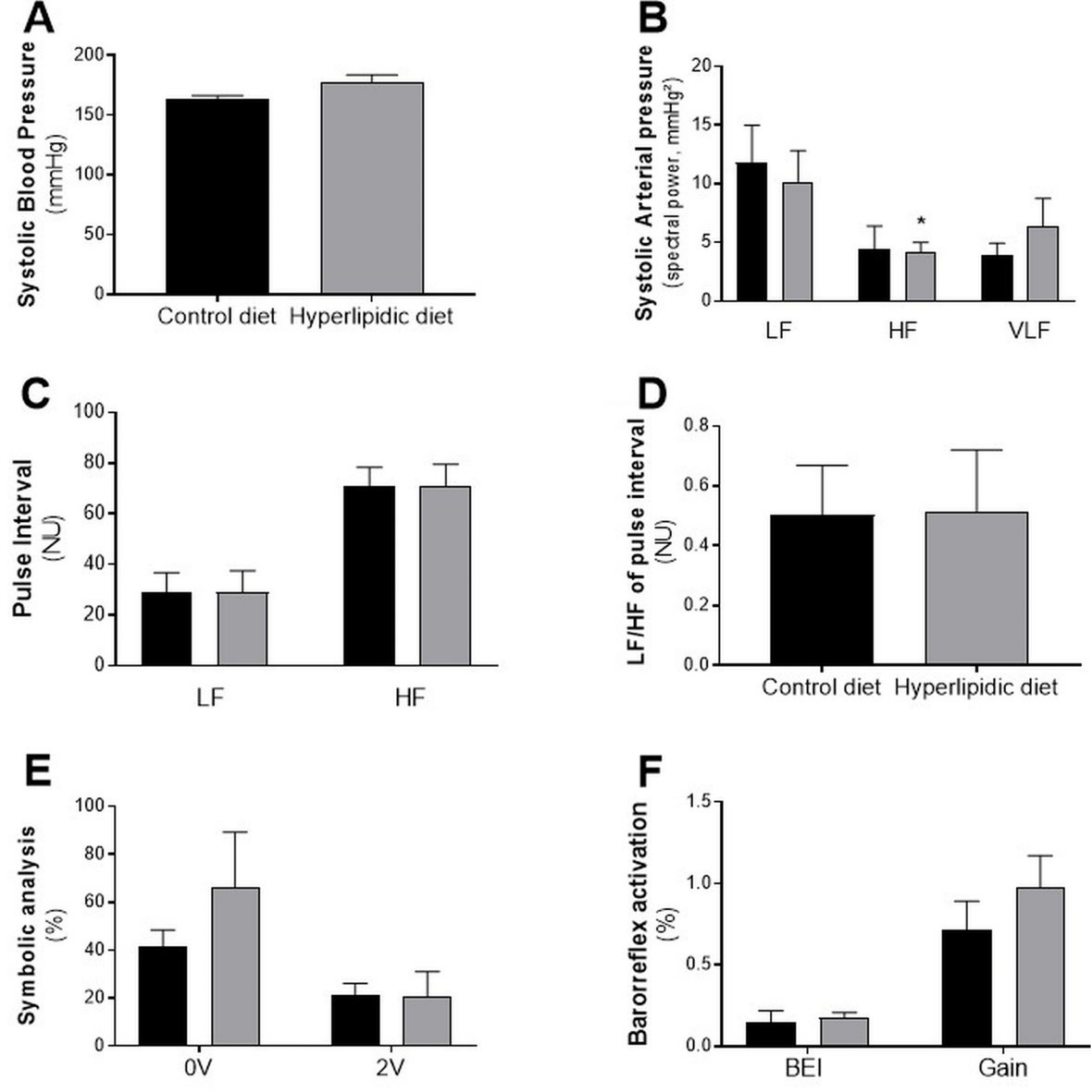
Analysis of spectral cardiac variability in 90-day Wistar rats on control and high-fat diets. **A**, Systolic blood pressure; **B**, low frequency (LF); high frequency (HF); and very low frequency (VLF) components of blood pressure; **C**, LF and HF components; **D**, LF/HF ratio of the cardiac interval; **E**, symbolic analysis of 0V and 2V components; **F**, Baroreflex Effectiveness Index (BEI) and baroreflex gain. Data are reported as mean±SE (n=4-5). *P<0.05; unpaired Student's *t*-test.

The effects of the experimental diet on cardiac function and autonomic modulation at 90 days is shown in Figure 2. As shown in Figure 2A and B, blood pressure increased considerably with the consumption of a diet high in saturated fat (Figure 2C; C=131.4±2.7 *vs* HL: 143.2±3.1 mmHg; P=0.0208) with no change in heart rate (Figure 2D). With respect to the effects of the HL diet on cardiac interval time domain, a reduction in the standard deviation of RR intervals (SDRR) (Figure 3C) was observed in the HL group (C=8.45±1.36 *vs* HL=4.84±0.30 ms; P=0.0127). The SD1 (Figure 3A), SD2 (Figure 3B), and root mean square successive difference (RMSSD) (Figure 3D) did not change. HL diet consumption did not affect systolic blood pressure (Figure 4A, C *vs* HL, P=0.09), but spectral analysis of systolic blood pressure revealed a reduction in HF waves in the HL group, as shown in Figure 4B (C=2.54±1.14 *vs* HL=4.97±0.25; P=0.0257), without changing the LF or VLF waves. The other variables, namely, the pulse interval (Figure 4C), LF/HF index (Figure 4D), symbolic analysis (Figure 4E), and baroreflex activation (Figure 4F), were not altered by the HL diet.

**Figure 5:**
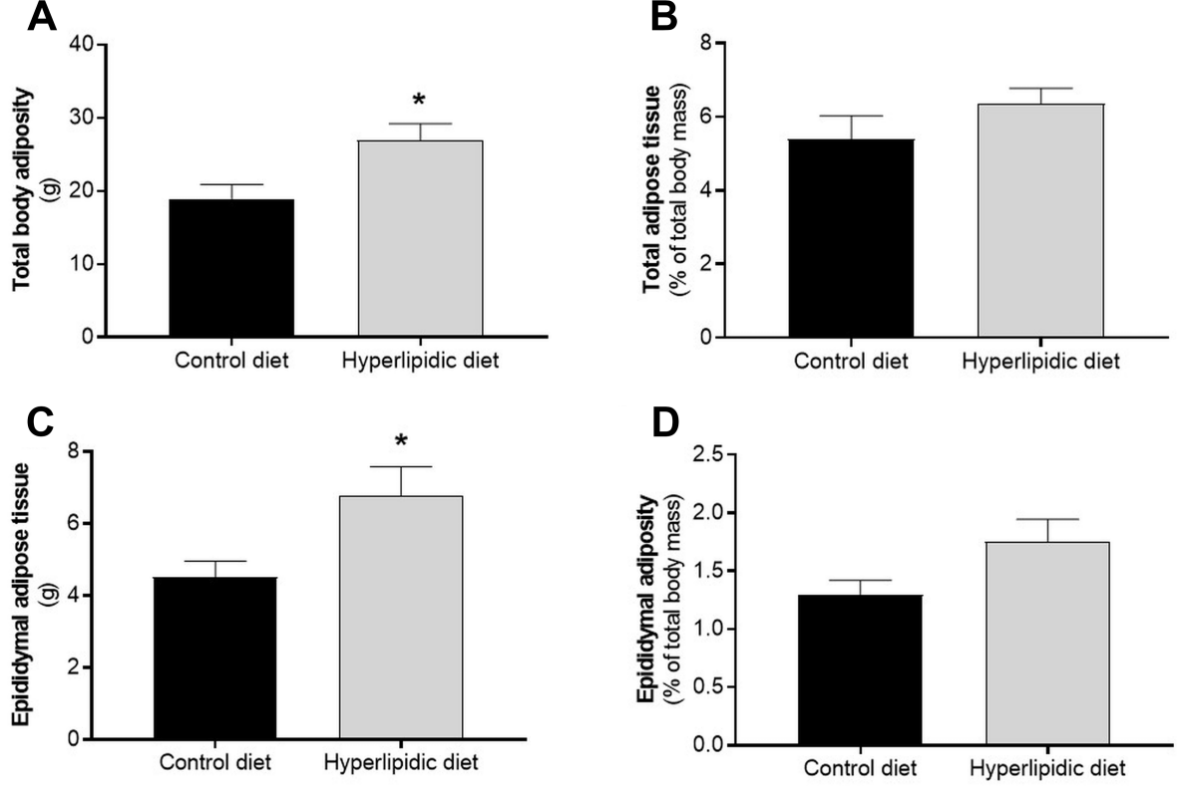
Body composition of Wistar rats on control and hyperlipidic diets rich in saturated fatty acids at 90 days. **A**, Total body adiposity; **B**, percent adipose tissue; **C**, epididymal adipose tissue; **D**, percent epididymal adipose tissue. Data are reported as means±SE (n=4-5). *P<0.05; unpaired Student's *t*-test.

The HL diet induced a greater accumulation of total body adiposity (Figure 5A; C=18.97±2.65 *vs* HL=26.98±2.69 g; P=0.0265) and epididymal adipose tissue (Figure 5C; C=4.52±0.44 *vs* HL=6.77±0.81 g; P=0.0399). However, when these values were corrected for the body weight of the animals, the differences were not maintained (Figure 5B and D).

**Figure 6:**
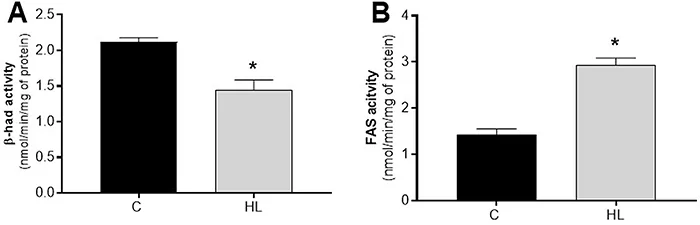
Activity of lipid metabolism enzymes in the epididymal adipose tissue of Wistar rats at 90 days on control (C) and hyperlipidic (HL) diets. **A**, β-HAD (β-hydroxyacyl-CoA dehydrogenase; **B**, FAS (fatty acid synthase). Data are reported as means±SE (n=4-5). *P<0.05; unpaired Student's *t*-test.

With respect to lipid metabolism in epididymal adipose tissue, compared with the C group, the HL group had a lower oxidation capacity (Figure 6A; C=2.12±0.06 *vs* HL=1.44±0.14 mmol/min per mg of protein) and greater fatty acid synthesis (Figure 6B; C=1.43±0.12 *vs* HL=2.94±0.14 mmol/min per mg of protein).

**Figure 7:**
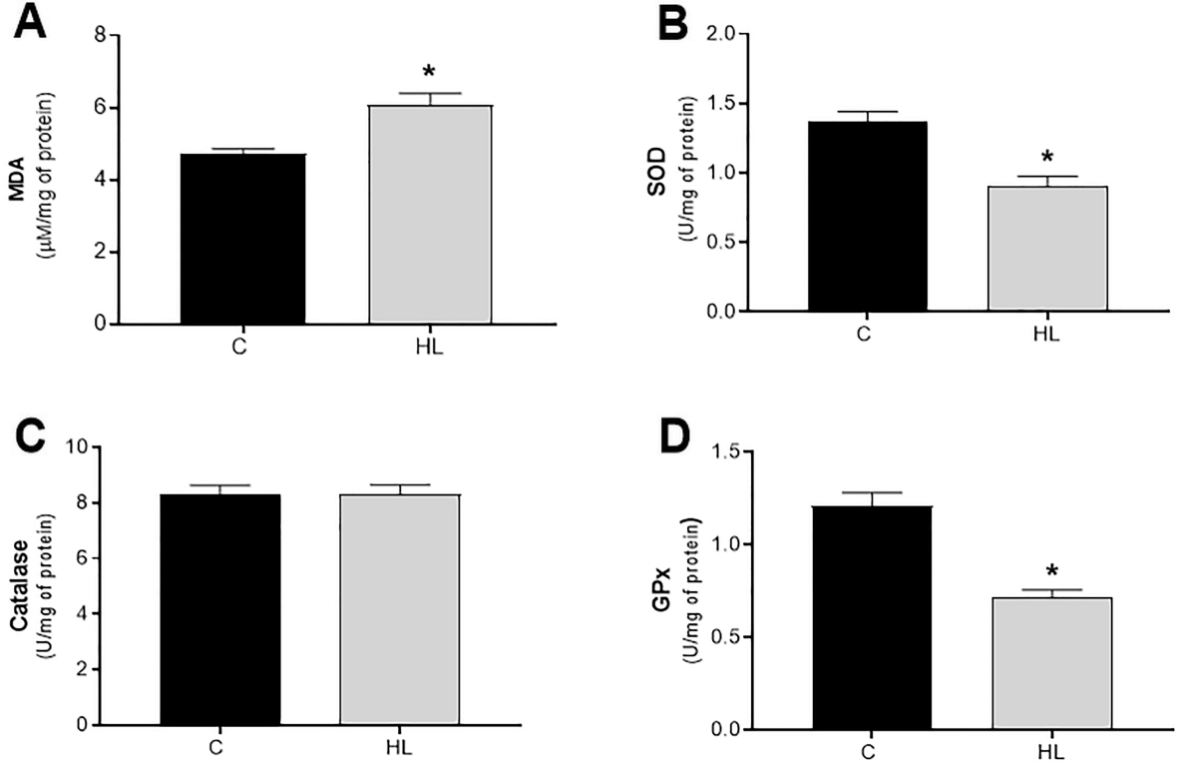
Antioxidant enzymes and oxidative stress markers in epididymal adipose tissue of Wistar rats on control (C) or hyperlipidic (HL) diets. **A**, Malondialdehyde (MDA); **B**, superoxide dismutase (SOD); **C**, catalase; **D**, glutathione peroxidase (GPx). Data are reported as mean±SE (n=4-5). *P<0.05; unpaired Student's *t*-test.

The results of the antioxidant enzymes and oxidative stress markers malondialdehyde (MDA), superoxide dismutase (SOD), catalase, and glutathione peroxidase (GPx) are reported in Figure 7. The HL group had higher levels of MDA (Figure 7A; C=4.71±0.15 *vs* HL=6.07±0.33; P=0.0025). In terms of antioxidant system activity, the HL diet decreased the activity of SOD (Figure 7B; C=1.37±0.08 *vs* HL=0.9±0.07; P=0.0011) and GPx (Figure 7D; C=1.21±0.07 *vs* HL=0.72±0.04; P=0.0001). Catalase activity did not differ between groups (Figure 7C).

## Discussion

Our study revealed that the offspring diet rich in saturated fatty acids altered mean arterial pressure and lipid metabolism profiles at 90 days without affecting autonomic control. Autonomic changes are likely to arise after the onset of metabolic dysfunction. These findings represent an initial step toward understanding adaptive responses to diets high in saturated fat, which may lead to subsequent metabolic changes. Westernized diets are frequently used to study the physiological and metabolic effects of excessive fat intake in early childhood (9).

However, food quality and macronutrient ratios vary widely among studies. Many of these diets contain high levels of fat (without discriminating its chemical structure) and protein deficits (5). In addition to lard, the lipid composition of margarine, despite having a lower saturated fatty acid profile than butter (17), is produced by the interesterification method (18). This process alters the stereoselective distribution of fatty acids, increasing the proportion of palmitic acid in the sn-2 position of triacylglycerols. The greater presence of this fatty acid in this position increases its bioavailability and favors its incorporation into chylomicrons and plasma lipoproteins. This redistribution is associated with increased low-density lipoprotein (LDL) cholesterol concentrations and reduced high-density lipoprotein (HDL) cholesterol concentrations that contribute to the imbalance of the lipid profile and the increase in atherogenic potential (19). Although no significant changes were observed in plasma lipid parameters, such as total cholesterol, triglycerides, and VLDL, in the present study, this does not exclude an increase in atherogenic potential, since the duration of high-fat dietary exposure may have been insufficient for changes in these markers to become detectable.

Although excessive fat consumption is associated with hyperphagia and fat accumulation (20), our animals did not show weight gain, fat accumulation, or increased adiposity after 12 weeks on a diet rich in saturated fats. These results contrast with those of a previous study (21), in which a Western diet, containing approximately 42% lipids, was compared to a high-fat diet with 60% lipids, which is commonly used to induce obesity in experimental rodent models. In general, high-fat diets, with between 20 and 60% of the total calories from lipids, have been widely used in diet-induced obesity models, mainly involving the use of lard and soybean oil as lipid sources. After 18 weeks of intervention, the animals subjected to these diets presented distinct metabolic phenotypes, indicating that the Western diet was more effective at inducing obesity and associated metabolic disorders.

This difference from our findings can be explained by relevant methodological differences, particularly the shorter duration of dietary exposure in the present study (9 *vs* 18 weeks) and the moderate lipid content of the high-fat diet used (32%), which is closer to that of the average human consumption and below the threshold commonly used to induce obesity in experimental models.

At the beginning of the experiment, animals fed the high-fat diet consumed fewer grams of food and fewer total calories, likely because of the higher energy density of the diet (4.46 *vs* 3.69 kcal/g in the control diet). Although total energy intake was lower in the HL group, this reduction occurred in parallel with a change in macronutrient intake, characterized by lower carbohydrate consumption and higher lipid consumption. Under these conditions, the absence of differences in body weight and visceral adiposity indicates that the reduction in total caloric intake was not sufficient to induce measurable changes in these parameters during the experimental period. Consequently, the EER was significantly greater in the HL group, likely reflecting the higher caloric density and lipid content of the diet, since high-fat diets are known to promote greater feed and metabolic efficiency (22), which may have contributed to maintain body weight control regardless of food intake.

Although systemic repercussions were not evident in these animals, changes in lipid metabolism and the redox system suggest possible long-term metabolic damage. Lipid overload, caused by excessive fat consumption, is a relevant factor in serum metabolic changes (23), especially with continuous consumption. Saturated fat is indicated by high serum triacylglycerols (TGs), and its removal becomes inefficient, resulting in a postprandial lipidemic state (24), which is associated with cardiovascular diseases (5). The marked increase in TG observed at 30 days of age in both groups likely reflected a transient metabolic adjustment during the postweaning period, characterized by changes in lipid handling and maturation of metabolic regulation, rather than a sustained dietary effect. The reduction observed at 90 days suggests metabolic adaptation over time.

In this study, there was no significant difference in any serum parameters between the groups, and the duration of exposure to the diet or the composition of saturated fatty acids was insufficient to promote certain types of serum overload, especially in relation to lipid profile, a result that has already been demonstrated in other studies (25).

Systolic, diastolic, and mean arterial pressures reflect different aspects of the cardiovascular function. Systolic pressure is influenced mainly by ventricular contractility and the elasticity of large arteries, whereas diastolic pressure represents peripheral vascular resistance and vascular tone during cardiac relaxation. On the other hand, mean arterial pressure provides an integrated measure of tissue perfusion and depends on both cardiac output and systemic vascular resistance. Therefore, the differences observed among these parameters in our study may indicate distinct hemodynamic adaptations to a high-fat diet.

An association of these results can be observed when examining the activity of key enzymes of lipid metabolism, which revealed greater oxidative capacity and reduced synthesis in animals fed a diet rich in saturated fatty acids. Excessive consumption of saturated fat is difficult to avoid in current Western diets (26). Saturated fats can alter lipid metabolism and disrupt homeostatic by increasing the expression of inflammatory pathways and decreasing the expression of anti-inflammatory pathways (27). First, metabolic signaling pathways such as c-Jun N-terminal kinase (JNK), nuclear factor kB (NF-kB), and protein kinase R are activated (28), which results in a low level of inflammatory cytokine production and a low-grade inflammatory response (29). Second, the metabolic imbalance caused by excess fat from the diet can lead to hyperplasia and hypertrophy of adipocytes, tissue remodeling, and an increase in free fatty acids, resulting in changes in the production of adipokines and an inflammatory response (30).

The role of the inflammatory process in inducing oxidative stress in this scenario cannot be ruled out, especially in adipose tissue. Although the relative amount of epididymal fat did not differ between groups, diets rich in saturated fatty acids have been shown to induce adipose tissue dysfunction characterized by inflammatory activation and impaired adipokine signaling. These alterations may occur independently of changes in absolute or relative fat mass and can compromise lipid handling and storage at the molecular level, representing early metabolic disturbances associated with an increased risk of obesity-related disorders (31). In addition, adipocyte hypertrophy is considered an important source of oxidative stress, and excessive production of ROS is involved in adipose tissue dysfunction (32).

In this work, the consumption of a higher content of saturated fatty acids increased the levels of MDA, which is positively correlated with visceral fat, as it is a predictor of lipid peroxidation and an indicator of cell damage through an increase in ROS (33). Furthermore, the high-fat diet impaired the antioxidant capacity by reducing the activity of SOD and GPx enzymes, which indicate oxidative stress induction in visceral adipose tissue.

These results are in line with other studies, where a diet rich in saturated fats increased the plasma and cardiac concentrations of MDA, which significantly increased when the calories in the diet came from fat (34). Other authors reported that high-fat diets with added fructose were able to reduce the activity of enzymes such as GPx and SOD, weakening antioxidant defense and intensifying oxidative stress and cellular damage in adipose tissue. This effect occurs because excess saturated fatty acids increase ROS generation through mitochondrial β-oxidation and NADPH oxidase activation, which inhibits the Nrf2/ARE pathway responsible for regulating the expression of antioxidant enzymes (35).

A hyperlipidic diet, in addition to being associated with increased ROS levels, is strongly associated with hypertension, an important risk factor for the development of coronary heart disease (36,37). Evidence suggests a strong relationship between an inadequate diet rich in saturated fatty acids, body fat levels, and blood pressure in both men and women; it also suggests that such diet is a contributing factor to high blood pressure (38).

In this study, animals fed a high-fat diet did not show any changes in the evaluated cardiovascular parameters, except for high mean arterial pressure, which clinically reflects the pressure in the aorta and great vessels, commonly called irrigation pressure (39). As explained in the literature, the aorta and large arteries have elastic walls that stretch during systole and increase blood flow during diastole; these movements are lost in the senile phase (40). The loss of this elasticity directly contributes to increased blood pressure levels, which could occur in animals that received a high-fat diet at an older age.

Furthermore, the present findings suggest that the metabolic and cardiovascular consequences of high-fat diets may vary depending on composition, duration of exposure, and lipid sources, as previously demonstrated in other experimental models.

In conclusion, the high-fat diet used in this study, although it did not affect the serum biochemical or morphological profile of the animals until 90 days of age, precipitated an increase in lipid peroxidation and a reduction in the activity of antioxidant enzymes, together with a significant increase in mean arterial pressure. These findings suggest that a diet rich in saturated fatty acids may be associated with greater induction of oxidative stress in visceral adipose tissue, which may precede the development of hypertension or other cardiovascular pathological conditions induced by the diet at an older age.

This study has several limitations, including the relatively short experimental duration and the absence of molecular and histological analyses that could further elucidate the mechanisms underlying these responses. Future studies with longer dietary exposure and assessment of additional tissues are therefore warranted to confirm and expand these findings.

## Data Availability

All data generated or analyzed during this study are included in this published article.
